# Two experts and a newbie: [^18^F]PARPi vs [^18^F]FTT vs [^18^F]FPyPARP—a comparison of PARP imaging agents

**DOI:** 10.1007/s00259-021-05436-7

**Published:** 2021-09-06

**Authors:** Sophie Stotz, Johannes Kinzler, Anne T. Nies, Matthias Schwab, Andreas Maurer

**Affiliations:** 1 Werner Siemens Imaging Center, Department of Preclinical Imaging and Radiopharmacy, Eberhard Karls University Tuebingen, Roentgenweg 15, 72076 Tuebingen, Germany; 2 Cluster of Excellence iFIT (EXC 2180) “Image Guided and Functionally Instructed Tumor Therapies”, Eberhard Karls University Tuebingen, Tuebingen, Germany; 3 Dr. Margarete Fischer-Bosch-Institute of Clinical Pharmacology, Stuttgart, and Eberhard Karls University Tuebingen, Tuebingen, Germany; 4 Departments of Clinical Pharmacology, and of Biochemistry and Pharmacy, Eberhard Karls University Tuebingen, Tuebingen, Germany

**Keywords:** PARP, PET imaging, Radiotracers, PARP inhibitors, Oncology

## Abstract

**Purpose:**

Imaging of PARP expression has emerged as valuable strategy for prediction of tumor malignancy. While [^18^F]PARPi and [^18^F]FTT are already in clinical translation, both suffer from mainly hepatobiliary clearance hampering their use for detection of abdominal lesions, e.g., liver metastases. Our novel radiotracer [^18^F]FPyPARP aims to bridge this gap with a higher renal clearance and an easily translatable synthesis route for potential clinical application.

**Methods:**

We developed a less lipophilic variant of [^18^F]PARPi by exchange of the fluorobenzoyl residue with a fluoronicotinoyl group and automated the radiosyntheses of the three radiotracers. We then conducted a comparative side-by-side study of [^18^F]PARPi, [^18^F]FPyPARP, and [^18^F]FTT in NOD.CB17-Prkdc^scid^/J mice bearing HCC1937 xenografts to assess xenograft uptake and pharmacokinetics focusing on excretion pathways.

**Results:**

Together with decent uptake of all three radiotracers in the xenografts (tumor-to-blood ratios 3.41 ± 0.83, 3.99 ± 0.99, and 2.46 ± 0.35, respectively, for [^18^F]PARPi, [^18^F]FPyPARP, and [^18^F]FTT), a partial shift from hepatobiliary to renal clearance of [^18^F]FPyPARP was observed, whereas [^18^F]PARPi and [^18^F]FTT show almost exclusive hepatobiliary clearance.

**Conclusion:**

These findings imply that [^18^F]FPyPARP is an alternative to [^18^F]PARPi and [^18^F]FTT for PET imaging of PARP enzymes.

**Supplementary Information:**

The online version contains supplementary material available at 10.1007/s00259-021-05436-7.

## Introduction

Critical for DNA repair [1], inhibition of poly(ADP ribose) polymerase (PARP) enzymes can be lethal for homologous recombination deficient (e.g., BRCA1/2 mutated) tumors due to the resulting inability to repair DNA single-strand breaks [2, 3]. The clinical potential of such PARP inhibitors is reflected by the efforts taken to develop different drugs targeting the PARP enzymes: since the first milestone, the clinical approval of olaparib [4], other inhibitors followed this path with the prominent candidates niraparib, veliparib, and rucaparib [5], and optimized next generation inhibitors (e.g., talazoparib/BMN 673) demonstrate even higher efficacy [6]. Competing with the cofactor NAD^+^ to interact with the enzyme’s binding site [7], PARP inhibitors both inhibit catalytical poly-ADP-ribosylation (PARylation) of target proteins but at the same time can hamper the function of the replication fork by trapping the enzyme on the DNA [8] in a not yet fully understood process, ultimately leading to enhanced susceptibility of the tumor for DNA-damaging agents [9–11]. Mainly applied as maintenance therapy after regular chemotherapy for recurrent cisplatin-sensitive BRCA-mutated ovarian cancer types [12, 13], the potential for the use of PARP inhibitors in non-BRCA-deficient tumors is evident as well [14].

Besides the therapeutic relevance of PARP inhibitors, non-invasive imaging of PARP with the aid of positron emission tomography (PET) possesses the potential to predict tumor malignancy since elevated PARP1 expression is associated with poor prognosis and lower survival rates in breast cancer and leukemia [15, 16]. Enhanced PARP expression levels are found in different tumor entities pointing towards a broad application spectrum of PARP imaging not only limited to BRCA-deficient tumors [17].

Within the last decade, several inhibitors of the enzyme were radiolabeled and preclinically evaluated for nuclear imaging [18–21] but two outstanding PARP imaging agents evolved to gold standards of PARP radiotracer development: [^18^F]PARPi [22] and [^18^F]FTT [23, 24]. Both radiotracers successfully reflect PARP1/2 expression levels and detect target engagement of PARP inhibitors [25–27]. The fluorescent variant PARPi-FL shows promising clinical data for its use to detect early oral squamous cell carcinomas [28, 29] and was already radiolabeled for use as dual-modality PET/optical imaging probe [30]. While the benefit of the radiotracers was assessed independently from each other, there is no direct comparison of the pharmacological properties in the same animal model available. Both radiotracers entered ([^18^F]FTT) [31] or successfully completed ([^18^F]PARPi) [32] clinical phase I trials; however, they suffer from mainly hepatobiliary clearance hampering their use for abdominal lesions such as liver metastases.

To expand the clinical scope, we present the alternative PARP imaging agent [^18^F]FPyPARP with a reduced logP value that is expected to shift the excretion route towards renal clearance. This was based on the empirical observations that the clearance pathway of a drug is often rather well predicted by only four physicochemical parameters including lipophilicity [33]. The new imaging agent [^18^F]FPyPARP indicates promise for improved non-invasive imaging of abdominal lesions. Furthermore, we compared the state-of-the-art [^18^F]PARPi and [^18^F]FTT to our novel radiotracer in the same animal model side-by-side, shedding light on clearance pathways and tumor uptake differences.

## Materials and methods

### Organic chemistry

All reagents and solvents were purchased from commercial suppliers and used without further purification if not stated otherwise. High-performance liquid chromatography (HPLC) columns were purchased from Phenomenex (Aschaffenburg, Germany). Electrospray ionization mass spectrometry (ESI–MS) was performed on a 1200 series HPLC system (Agilent, Waldbronn, Germany) equipped with a 6120 quadrupole mass spectrometer. A gradient of acetonitrile (MeCN) in 0.1% aqueous formic acid on a Luna C18(2) column (50 mm × 2 mm, 100 Å, 5 μm) was used for separation. Nuclear magnetic resonance (NMR) spectra were acquired using a 600-MHz Avance III spectrometer (Bruker Biospin, Ettlingen, Germany).

### Synthesis of nonradioactive PARPi

For synthesis of 4-(4-fluorobenzoyl)piperazine-1-carbonyl)benzyl)phthalazine-1(2*H*)-one (PARPi), 4-fluorobenzoic acid (1 eq, 0.273 mmol, 0.03825 g) and 4-(4-fluoro-3-(piperazine-1-carbonyl)benzyl)phthalazine-1(2*H*)-one (1 eq, 0.273 mmol, 0.1 g, AB478852, abcr, Karlsruhe, Germany) were dissolved in 20 ml dichloromethane (DCM) and 475 µl diisopropylethylamine (DIPEA, 10 eq, 2.73 mmol) was added. After addition of 2-(1*H*-benzotriazole-1-yl)-1,1,3,3-tetramethylaminium tetrafluoroborate (TBTU, 1.2 eq, 0.328 mmol, 0.105 g), the reaction was stirred overnight. The solvent was evaporated and a fraction of the product was purified by preparative HPLC on a Luna C18(2) column (250 mm × 10 mm, 100 Å, 10 μm) on a 1260 Infinity HPLC system (Agilent) with 60% aqueous, 0.1% trifluoroacetic acid (TFA), and 40% MeCN. ^1^H NMR (600 MHz, chloroform-*d*) δ 10.57 (s, 1H), 8.47 (d, *J* = 7.8, 1H), 7.83–7.78 (m, 2H), 7.75–7.73 (m, 1H), 7.44–7.41 (m, 2H), 7.36–7.30 (m, 2H), 7.13–7.10 (m, 3H), 4.30 (s, 2H), 3.75–3.36 (m, 8H). ESI–MS (m/z) calc. [M-H]^−^ 487.16, found 487.2.

### Synthesis of [^18^F]FPyTFP precursor

*N,N,N*-Trimethyl-5-((2,3,5,6-tetrafluorophenoxy)carbonyl)pyridine-2-amminium trifluoromethanesulfonate was synthesized according to literature [34]. Instead of trimethylamine gas as stated in the procedure, 2 M trimethylamine in tetrahydrofuran (THF) was used. TFP ester synthesis had a yield of 66% while the combined yield of the trimethylamine incorporation and counterion exchange steps (TMSOTf) was 58%. ^1^H NMR (600 MHz, acetonitrile-*d*_3_) δ 9.34 (dd, *J* = 2.3, 0.8 Hz, 1H), 8.85 (dd, *J* = 8.7, 2.3 Hz, 1H), 8.08 (dd, *J* = 8.7, 0.8 Hz, 1H), 7.43 (tt, *J* = 10.5, 7.3 Hz, 1H), 3.60 (s, 9H). ESI–MS (m/z) calc. M^+^ 329.09, found 329.1.

### Synthesis of nonradioactive FPyPARP

4-(4-Fluoro-3-(4-(6-fluoronicotinoyl)piperazine-1-carbonyl)benzyl)phthalazine-1(2*H*)-one was synthesized by dissolving 4-(4-fluoro-3-(piperazine-1-carbonyl)benzyl)phthalazine-1(2*H*)-one (1 eq, 0.59 mmol, 0.2 g) and 6-fluoronicotinic acid (1 eq, 0.59 mmol, 0.77 g) in 10 ml DCM, adding 1-ethyl-3-(3-dimethylaminopropyl)carbodiimide (EDC, 1 eq, 0.59 mmol, 0.119 g) and stirring the reaction overnight. The solvent was evaporated and a portion of the product was purified by column chromatography with ethyl acetate containing 1% triethylamine (yield: 34%). ^1^H NMR 600 MHz, chloroform-*d*) δ 11.11 (s, 1H), 8.48–8.43 (m, 1H), 8.30 (s, 1H), 7.92–7.86 (m, 1H), 7.80–7.73 (m, 2H), 7.73–7.69 (m, 1H), 7.34 (dd, *J* = 6.2, 2.0 Hz, 2H), 7.09–6.94 (m, 2H), 4.29 (s, 2H), 4.05–3.19 (m, 8H). ESI–MS (m/z) calc. [M-H]^−^ 488.16, found 488.1.

### Synthesis of [^18^F]FTT tosylate precursor

1-(4-Hydroxyphenyl)-8,9-dihydro-2,7,9a-triazabenzo[cd]azulen-6(7*H*)-one was synthesized according to the patent WO2018218025. Of this compound, 0.025 g (1 eq, 0.09 mmol) was dissolved in 5 ml MeCN together with 0.167 g ethylene bistosylate (5 eq, 0.45 mmol) and 0.018 g potassium carbonate (1.5 eq, 0.018 mmol) and stirred in a sealed container overnight. The solvent was evaporated and the product was purified by flash column chromatography using 0–5% methanol in DCM (yield: 36%). ^1^H NMR (600 MHz, DMSO-*d*_6_) δ 8.45 (t, *J* = 5.8 Hz, 1H), 7.87 (d, *J* = 7.9 1H), 7.87–7.80 (m, 3H), 7.83–7.76 (m, 2H), 7.49 (d, *J* = 8.1 Hz, 2H), 7.34 (t, *J* = 7.8 Hz, 1H), 7.07–7.01 (m, 2H), 4.44 (m, 2H), 4.41–4.37 (m, 2H), 4.30–4.25 (m, 2H), 3.53 (m, 2H), 2.42 (s, 3H). ESI–MS (m/z) calc. [M + H]^+^ 478.14, found 478.0.

### Synthesis of nonradioactive FTT

4-(2-Fluoroethoxy)benzoyl chloride was produced by activating 0.25 g of 4-(2-fluoroethoxy)benzoic acid (1 eq, 1.36 mmol) with 1 ml thionyl chloride (10 eq, 13.7 mmol) for 3 h at 85 °C. The vacuum-dried compound was added without further purification to 9-amino-1,2,3,4,-tetrahydro-5*H*-1,4,-benzodiazepin-5-one (1 eq, 0.057 g) dissolved in 5 ml DCM and 5 ml pyridine and stirred overnight. The solvents were evaporated and the residue was dissolved in 50 ml methanol. One milliliter methanesulfonic acid was used to induce cyclization for 2 h under reflux at 75 °C. The solvent was again evaporated, dissolved in 75 ml ethyl acetate, and washed with 50 ml of each saturated Na_2_CO_3_, water, and brine. After drying with MgSO_4_, the solvent was evaporated and a portion of the product was purified by flash column chromatography using 15% methanol in ethyl acetate (yield: 15%). ^1^H NMR (600 MHz, DMSO-*d*_6_) δ 8.36 (t, *J* = 5.8 Hz, 1H), 7.79 (dd, *J* = 7.9, 1.1 Hz, 1H), 7.76 (dd, *J* = 7.6, 1.1 Hz, 1H), 7.76–7.71 (m, 2H), 7.26 (t, *J* = 7.8 Hz, 1H), 7.11–7.06 (m, 2H), 4.77–4.73 (m, 1H), 4.69–4.65 (m, 1H), 4.39–4.34 (m, 2H), 4.31–4.27 (m, 1H), 4.26–4.22 (m, 1H), 3.45 (q, *J* = 5.7, 4.9 Hz, 2H). ESI–MS (m/z) calc. [M + H]^+^ 326.12, found 326.1.

### Radiolabeling

All radiosyntheses ([^18^F]PARPi, [^18^F]FPyPARP, and [^18^F]FTT) were automated on a modified TRACERlab radiochemical synthesizer (GE Healthcare, Uppsala, Sweden) and [^18^F]fluoride was produced on a medical cyclotron (PETtrace 800, GE Healthcare) using the ^18^O(p,n)^18^F nuclear reaction. The HPLC signal at 254 nm was used for calculation of the carrier concentration. Content of free [^18^F]fluoride was analyzed by thin layer chromatography (TLC, PolyGram Sil G/UV254, Macherey–Nagel, Dueren, Germany; eluent, 100% ethyl acetate) and was below 0.5%.

### [^18^F]PARPi

[^18^F]Fluoride was trapped on an ion exchange cartridge (Sep-Pak Plus Light QMA Carb, Waters, Waltham, MA, USA) preconditioned with 10 ml 1 M NaHCO_3_ and 10 ml H_2_O and eluted with 2 ml MeCN containing 4% H_2_O, 9.5 mg Kryptofix 2.2.2, and 1.7 mg K_2_CO_3_ and azeotropically dried under a stream of helium. Successively, 5 mg ethyl-4-trimethylammonium benzoate triflate (SFB precursor) in 0.5 ml dimethylsulfoxide (DMSO) (8 min at 120 °C), 15 mg *t*BuOK in 0.5 ml DMSO (5 min at 120 °C), and 30 mg *N,N,N′,N′*-tetramethyl-*O*-(*N*-succinimidyl)uronium tetrafluoroborate (TSTU) in 2 ml MeCN (5 min at 100 °C) were added to the reaction vial. After cooling to 50 °C and addition of 1 ml 5% acetic acid to prevent hydrolysis, the reaction was diluted in 25 ml H_2_O and trapped on a Sep-Pak Plus Light C18 cartridge (Waters) preconditioned with 10 ml ethanol and 10 ml H_2_O. The synthon [^18^F]SFB was eluted back in the reactor vial with 0.5 ml DMF containing 10 mg 4-(4-fluoro-3-(piperazine-1-carbonyl)benzyl)phthalazin-1(2*H*)-one (AB478852, abcr) and 40 µl DIPEA and heated for 10 min to 120 °C. Two milliliter 0.1% TFA containing 30% MeCN was added and the reaction mixture was purified on a Luna C18(2) column (250 mm × 10 mm, 100 Å, 10 µm) with 30% aqueous MeCN containing 0.1% TFA and a flow of 7 ml/min. The product peak (retention time 15–20 min, detected using an online radioactivity detector) was collected, diluted in 50 ml H_2_O, and trapped on a Sep-Pak Plus Light C18 cartridge (Waters) preconditioned with 10 ml ethanol and 10 ml H_2_O. The product was eluted into the product vial with 0.5 ml ethanol, followed by 4.5 ml phosphate-buffered saline (PBS). Quality control was performed on a Luna C18(2) column (250 mm × 4.6 mm, 100 Å, 5 µm) with 55% 0.1% aqueous TFA and 45% MeCN with a flow rate of 1 ml/min on a 1260 Infinity II HPLC system (Agilent) with radioactivity detector. Data on starting activity, radiochemical yield, and molar activity of the individual syntheses are provided in Supplementary Table 1.

### [^18^F]FPyPARP

[^18^F]Fluoride was trapped on an ion exchange cartridge (Sep-Pak Plus Light QMA Carb, Waters) preconditioned with 10 ml 1 M NaHCO_3_ and 10 ml H_2_O and eluted with 0.075 M tetrabutylammonium hydrogen carbonate (TBAHCO_3_) and MeCN (50:50) producing [^18^F]TBAF which was dried azeotropically under a helium stream. Five milligram [^18^F]FPyPARP precursor in 500 µl *tert*-butanol (*t*-BuOH) and MeCN (8:2) was added to the reactor for 10 min at 40 °C, followed by the addition of 4-(4-fluoro-3-(piperazine-1-carbonyl)benzyl)phthalazin-1(2*H*)-one (AB478852, abcr) in 500 µl MeCN containing 40 µl DIPEA and incubation for 10 min at 120 °C. Three milliliter water were added and the reaction mixture was purified on a Luna C18(2) column (250 mm × 10 mm, 100 Å, 10 µm) with 25% aqueous MeCN containing 0.1% TFA and a flow rate of 7 ml/min. The product peak (retention time 15–20 min, detected using an online radioactivity detector) was collected, diluted in 50 ml H_2_O, and trapped on a Sep-Pak Plus Light C18 cartridge (Waters) preconditioned with 10 ml ethanol and 10 ml H_2_O. The product was eluted into the product vial with 0.5 ml ethanol, followed by 4.5 ml PBS. Quality control was performed on a Luna C18(2) column (250 mm × 4.6 mm, 100 Å, 5 µm) with 65% 0.1% aqueous TFA and 35% MeCN with a flow rate of 1 ml/min on a 1260 Infinity II HPLC system (Agilent) with radioactivity detector. Data on starting activity, radiochemical yield, and molar activity of the individual syntheses are provided in Table S1.

#### [^18^F]FTT

[^18^F]Fluoride was trapped on an ion exchange cartridge (Sep-Pak Plus Light QMA Carb, Waters) preconditioned with 10 ml 1 M NaHCO_3_ and 10 ml H_2_O and eluted with 2 ml MeCN containing 4% H_2_O, 9.5 mg Kryptofix 2.2.2, and 1.7 mg K_2_CO_3_ which was subsequently dried azeotropically under a helium stream. One milligram [^18^F]FTT precursor in 750 µl DMF was added to the reactor and the reaction mixture was stirred for 10 min at 105 °C. Three milliliter 17% MeCN in 0.1% aqueous TFA was added and the reaction mixture was purified on a Luna C18(2) column (250 mm × 10 mm, 100 Å, 10 µm) with 17% aqueous MeCN containing 0.1% TFA and a flow rate of 5 ml/min. The product peak (retention time 9–10 min, detected using an online radioactivity detector) was collected, diluted in 50 ml H_2_O, and trapped on a Sep-Pak Plus Light C18 cartridge (Waters) preconditioned with 10 ml ethanol and 10 ml H_2_O. The product was eluted into the product vial with 0.5 ml ethanol, followed by 4.5 ml PBS. Quality control was performed on a Luna C18(2) column (250 mm × 4.6 mm, 100 Å, 5 µm) with 0.1% aqueous TFA containing 32% MeCN with a flow rate of 1 ml/min on a 1260 Infinity II HPLC system (Agilent) with radioactivity detector. Data on starting activity, radiochemical yield, and molar activity of the individual syntheses are provided in Table S1.

#### Serum stability analysis

Serum stability was assessed by mixing [^18^F]FPyPARP solution 1:1 with C57BL/6 J mouse or human (blood type AB + , Sigma-Aldrich, Steinheim, Germany) serum. After 0-, 30-, 60-, 120-, and 240-min incubation at 37 °C, samples were drawn and the proteins were precipitated by adding ice-cold MeCN to a final concentration of 50%. The supernatant after centrifugation (12,100 × *g*, 90 s) was analyzed by HPLC as described for radiotracer quality control.

#### Experimental logP and logD determination

Water (logP), PBS (logD), and 1-octanol were saturated with the respective other phase for 24 h before the experiment. One microliter radiotracer solution (0.4 MBq) was added to a mixture of 500 µl water or PBS and 500 µl 1-octanol. After thorough shaking, the suspension was shortly centrifuged for phase separation, samples were drawn from each phase, and radioactivity concentration was measured in a gamma counter (WIZARD2, PerkinElmer, Waltham, MA, USA).

#### Cell culture

Human breast carcinoma cells (HCC1937, ACC513) were purchased from the German Collection of Microorganisms and Cell Cultures (DSMZ GmbH, Braunschweig, Germany) and cultured in Roswell Park Memorial Institute (RPMI) 1640 medium supplemented with 16% fetal calf serum (FCS), 100 U/ml penicillin, and 100 µg/ml streptomycin at 37 °C under 5% CO_2_ atmosphere. The absence of mycoplasma infection was confirmed by PCR analysis in monthly intervals.

#### Western blot

HCC1937 cells were lysed using radioimmunoprecipitation assay (RIPA) buffer (Thermo Scientific, Waltham, MA, USA) containing protease inhibitor (cOmplete Mini, EDTA-free, Roche, Basel, Switzerland) and protein concentration was determined using a commercial bicinchoninic acid (BCA) assay kit according to the manufacturer’s instruction (Thermo Scientific). Samples containing 40 µg of protein were boiled in reducing loading buffer and discontinuous Laemmli SDS-PAGE was performed with gels containing 12% polyacrylamide using the Mini-PROTEAN Tetra system (Bio-Rad, Hercules, CA, USA). The proteins were transferred on a polyvinylidene fluoride membrane using the same system and blocked for 1 h with Odyssey blocking buffer (Li-Cor, Lincoln, NE, USA) at room temperature after which the blot was incubated at 4 °C overnight in PBS with primary antibodies (1: mouse anti-PARP-1 IgG, clone C-2–10, BML-SA250-0050, Enzo life sciences, New York, NY, 1:3000; 2: rabbit anti-β-actin IgG, clone 13E5, Cell Signaling Technology, Danvers, MA, USA, 1:3000). The next day, the blot was washed twice with PBS-T, incubated with secondary antibodies (1: goat anti-mouse IgG, IRDye 680 RD, 1:7,000; 2: goat anti-rabbit IgG, IRDye 800 CW, 1:7,000, Li-cor) for 1 h at room temperature, and imaged after washing twice with PBS-T on an Odyssey Sa Infrared Imaging System (Li-Cor).

#### In vitro radiotracer uptake and acid wash

HCC1937 cells (0.2 × 10^6^) were incubated in 96-well filter plates (MADVN6550, Merck Millipore, Darmstadt, Germany) with 60 µl of a 0.4 MBq/ml radiotracer solution containing either 2.5 µl DMSO as vehicle or 2.5 µl 10 mM olaparib to a final concentration of 25 µM as blocking control. After 30 min of incubation at 37 °C, the cells were washed by vacuum filtration of medium through the plate (2 × 100 µl followed by 2 × 200 µl) and the filters were transferred into tubes using a commercial punch kit (MAMP09608, Merck) and measured in a gamma counter (Wizard 2, PerkinElmer, Waltham, MA, USA).

For the acid wash, cells were distributed in 96-well filter plates as described before and incubated with the radiotracer solution for 30 min at 37 °C. After an initial wash with 100 µl medium, cells were either washed twice (1: 100 µl, 2: 200 µl) with medium or with glycine–HCl in PBS (50 mM, pH 2.8) followed by a final wash with 200 µl medium and measured in a gamma counter.

#### PET/MR imaging and ex vivo biodistribution

All animal experiments were performed according to the German animal welfare act and approved by the local authorities (Regierungspräsidium Tübingen, R3/18). Animals were housed in individually ventilated cages (IVCs, 5 mice per cage) with bedding and enrichment, and food and water was provided ad libitum. Animals were kept under isoflurane anesthetic (1.5% in pure oxygen, 1.5 l/min) during the experiments. In 1:1 ice-cold Matrigel (Thermo Scientific)/PBS, 1 × 10^7^ cells were injected subcutaneously in the right shoulder area of 7-week-old female NOD.CB17-Prkdc^scid^/J mice (*n* = 10 per tracer). After the xenografts reached a suitable size (302 ± 152 mm^3^), mice were injected with 12.45 ± 0.87 MBq, 12.68 ± 0.39 MBq, and 12.94 ± 0.45 MBq, respectively, of [^18^F]PARPi, [^18^F]FPyPARP, and [^18^F]FTT and subjected to 1-h dynamic (*n* = 7 per tracer) or 10-min static (*n* = 3 per tracer, 1-h resting uptake) PET imaging (Inveon D-PET, Siemens, Knoxville, TN, USA) with subsequent magnetic resonance (MR) anatomical scans using a 7-T Biospec 70/30 USR (Clinscan, Bruker Biospin MRI GmbH) and a T2-weighted spin echo sequence. Five mice of each dynamically acquired group underwent a second, 10-min static PET scan 2-h post-injection (p.i.). Mice were sacrificed by cervical dislocation, the collected organs were weighed, and the tissue uptake was determined by gamma-counting (WIZARD2). Liver and kidney tissue was frozen in Tissue-Tek (Labtech, East Sussex, Britain) and 20-µm slices were prepared for autoradiography. A storage phosphor screen (Molecular Dynamics, Caesarea, Israel) was exposed to the sections for 18 h and scanned at a resolution of 100 µm/px with a STORM phosphor imager (Molecular Dynamics). PET image reconstruction and correlation with MR images was performed with Inveon Acquisition Workplace and Inveon Research Workplace, respectively, using a user-defined dynamic framing and an ordered subset expectation maximization (OSEM3D) algorithm. Regions of interest (ROIs) were drawn according to the acquired MR images and co-registered with the PET data to obtain time-activity curves (TACs).

#### Ex vivo immunofluorescence microscopy

Immunofluorescence microscopy was performed by the Department of Dermatology at the University of Tuebingen, Germany. Sections of paraffin-embedded xenografts were blocked with donkey serum for 30 min and incubated with primary antibody overnight (rabbit polyclonal anti-human PARP1 ab74290, Abcam, Cambridge, UK, 1:50). After washing, the sections were incubated for 1 h at room temperature with secondary antibody (Cy3-conjugated donkey anti-rabbit IgG 711–166-152, Dianova, Hamburg, Germany, 1:250). Nuclei were stained with YO-PRO-1 iodide (Thermo Fisher Scientific) for 5 min according to the manufacturer’s instructions; the samples were subsequently mounted with Mowiol (Sigma-Aldrich) and imaged on a LSM 800 microscope (Carl Zeiss, Oberkochen, Germany).

#### Statistical analyses

Statistical analyses are represented as mean values ± standard deviation. Analyses were performed using GraphPad Prism 9 and non-parametric *t* tests (comparison of two groups) and one-way ANOVA (comparison of more than two groups). Blood half-life was calculated using a two-phase decay fit in GraphPad Prism 8. *p* values < 0.05 were considered statistically significant according to the software (*p < 0.05, **p < 0.01, ***p < 0.001, ****p < 0.0001).

#### Vector graphics

All vector graphics in this work were created with Inkscape 0.92.

## Results

### Radiotracer syntheses

**Fig. 1:**
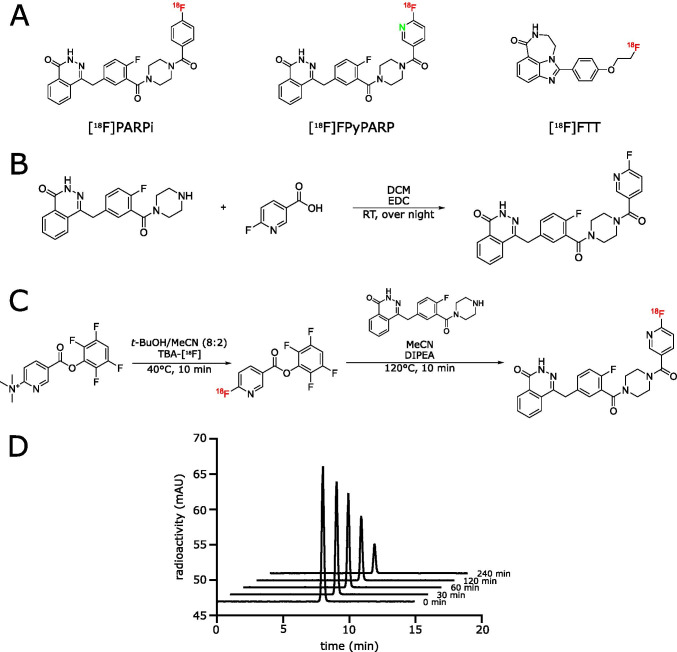
Synthesis routes for [^18^F]FPyPARP and the nonradioactive FPyPARP and serum stability analysis. **A** Structures of the three radiotracers compared in this study: [^18^F]PARPi, [^18^F]FPyPARP, and [^18^F]FTT. **B** Organic synthesis pathway of the nonradioactive FPyPARP. **C** Radiolabeling strategy for [^18^F]FPyPARP. **D** Serum stability measurements in mouse serum

The radiolabeling procedures of three PARP radiotracers—based on the clinically approved PARP inhibitors olaparib ([^18^F]PARPi and [^18^F]FPyPARP, only differing in two atoms) and rucaparib ([^18^F]FTT) (Fig. 1A)—were automated in our laboratories.

The [^18^F]PARPi synthesis was adapted from Carney et al. [22] using [^18^F]SFB as a synthetic intermediate to ease automation, resulting in 6.6 ± 3.2% radiochemical yield (RCY) in 94 ± 6 min synthesis time, radiochemical purity of > 95%, and a molar radioactivity of 79 ± 56 GBq/µmol (*n* = 17, representative HPLC analysis Supplementary Fig. 1). The novel [^18^F]FPyPARP is structurally following the idea of [^18^F]PARPi but using fluoronicotinic acid instead of fluorobenzoic acid as prosthetic group to decrease the clogP ([^18^F]PARPi: 3.36 vs [^18^F]FPyPARP: 2.49; [^18^F]FTT: 3.09) with the intention to foster renal clearance. The nonradioactive compound was readily synthesized by conjugating 4-(4-fluoro-3-(piperazine-1-carbonyl)benzyl)phthalazine-1(2*H*)-one with fluoronicotinic acid in analogy to the synthesis of nonradioactive PARPi (Fig. 1B). [^18^F]FPyPARP was synthesized in a two-step-one-pot reaction via the synthon [^18^F]FPyTFP with subsequent addition of 4-(4-fluoro-3-(piperazine-1-carbonyl)benzyl)phthalazine-1(2*H*)-one (Fig. 1C) resulting in a RCY of 9.9 ± 6.7% in 71 ± 4 min synthesis time, radiochemical purity of > 95%, and a molar radioactivity of 31 ± 12 GBq/µmol (*n* = 8, representative HPLC analysis Supplementary Fig. 2). [^18^F]FTT was automated in a one-pot synthesis by direct radiofluorination of the tosylate precursor that gave 9.5 ± 4% RCY in 51 ± 2.5 min synthesis time, radiochemical purity of > 95%, and 129 ± 38 GBq/µmol molar radioactivity (*n* = 4, representative HPLC analysis Supplementary Fig. 3). Additional information on the individual radiosyntheses is listed in Supplementary Table 1.

Serum stability analysis of [^18^F]FPyPARP was conducted in C57BL/6 J mouse serum and human serum showing no significant radiometabolites and good radiotracer stability over 240 min (Fig. 1D, Supplementary Fig. 4). In addition, logP ([^18^F]PARPi: 2.09, [^18^F]FPyPARP: 1.16; [^18^F]FTT: 1.10) and logD ([^18^F]PARPi: 2.09, [^18^F]FPyPARP: 1.16; [^18^F]FTT: 1.94) values were experimentally determined to validate the calculated logP values.

### In vivo radiotracer comparison

**Fig. 2:**
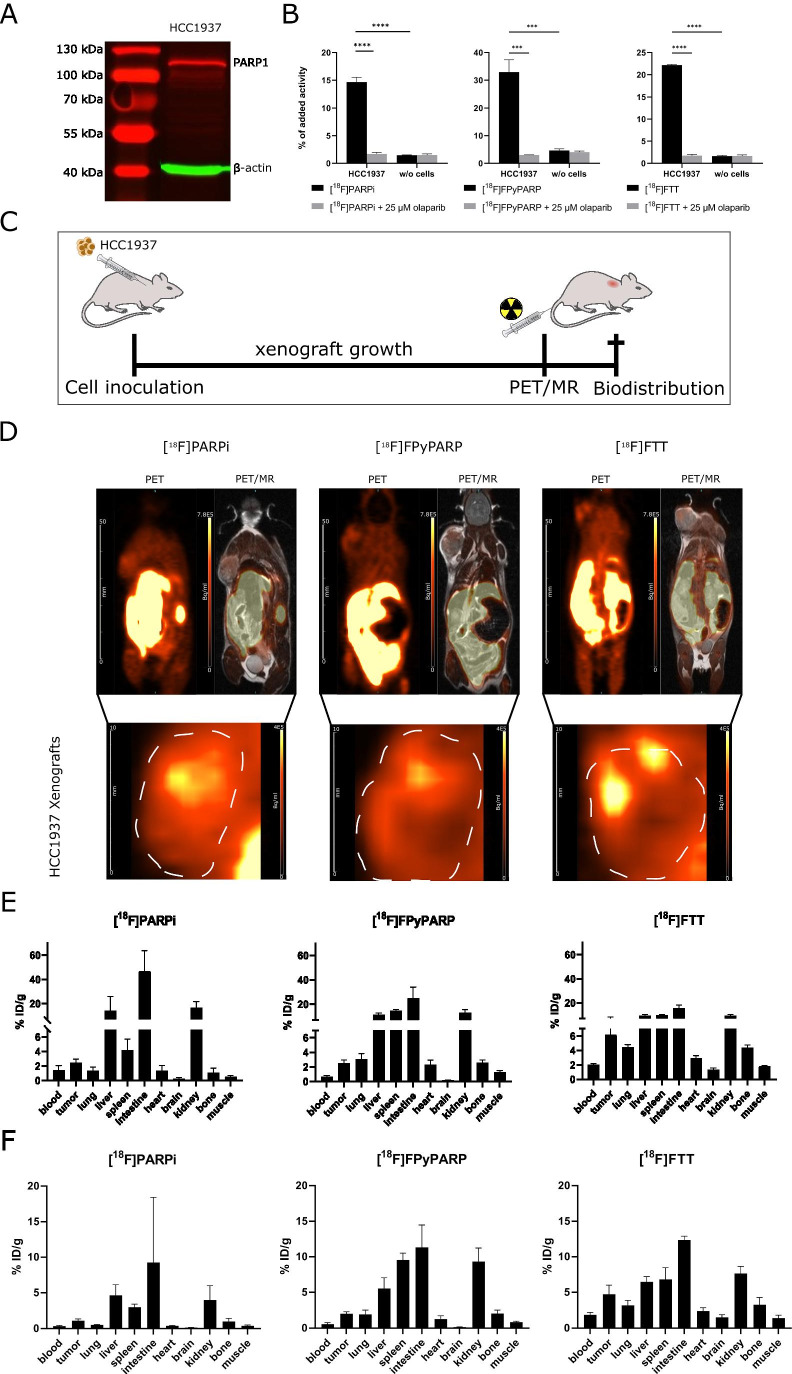
In vitro characterization of PARP expression in HCC1937 cells and in vivo analyses of [^18^F]PARPi, [^18^F]FPyPARP, and [^18^F]FTT. **A** PARP Western blot of HCC1937 cells using β-actin as loading control. **B** [^18^F]PARPi, [^18^F]FPyPARP, and [^18^F]FTT uptake and olaparib blocking in HCC1937 cells. **C** Schematic depiction of the in vivo experimental time line. **D** Representative PET/MR images of mice injected with either [^18^F]PARPi, [^18^F]FPyPARP, and [^18^F]FTT. A close-up on the xenografts is provided below. As time point, the last 10 min of the dynamic PET scans was chosen (minutes 50–60). Size bars represent 50 mm (whole body) or 10 mm (xenografts) and color-coded intensity bars range from 0 to 7.8 × 10^5^ Bq/ml (whole body) or 0 to 4 × 10^5^ Bq/ml (xenografts). **E** Ex vivo biodistribution analysis of the three radiotracers 1.5 h p.i. **F** Ex vivo biodistribution analysis of the three radiotracers 2.5 h p.i

The chosen HCC1937 cell model was already successfully applied in [^18^F]FTT radiotracer evaluation and thus used in this work as a standard xenograft model. To verify PARP expression of the cell line, HCC1937 cells were analyzed by Western blotting showing a prominent band at the expected size of PARP1 (Fig. 2A). In vitro tracer uptake experiments with using olaparib as blocking controls were conducted to ensure specificity of the radiotracer uptake in the cell model displaying significant (*p* < 0.001) and quantitative blocking of the radiotracer uptake (Fig. 2B). No reduction in the radiotracer signal was observed when the cells were washed with an acidic buffer instead of only medium (Supplementary Fig. 5).

**Fig. 3:**
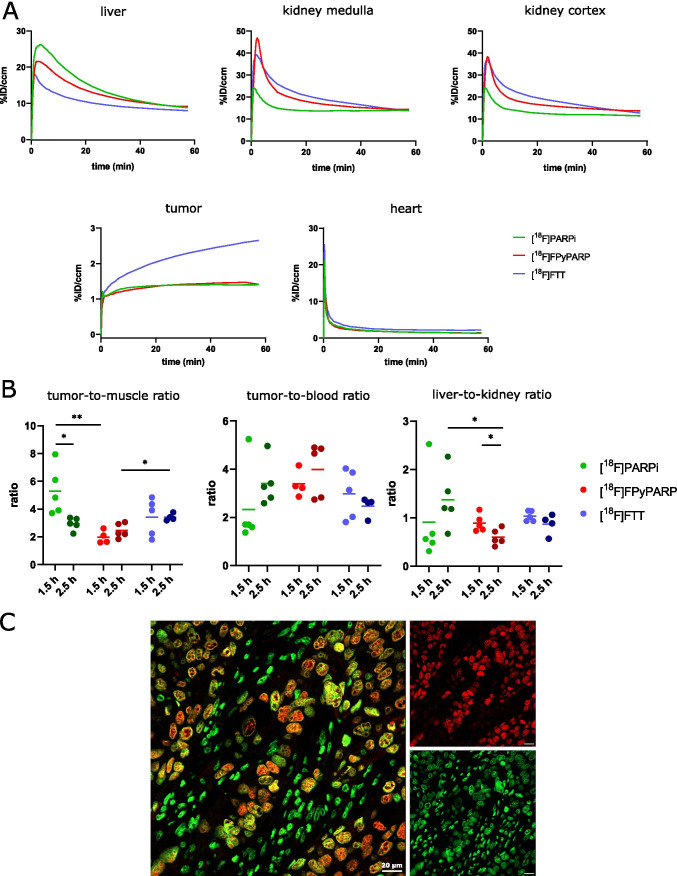
Time-activity curves and tumor-to-muscle, tumor-to-blood, and liver-to-kidney ratios in comparison. **A** Mean TACs of the liver, kidney (separated in medulla and cortex), tumor, and heart of [^18^F]PARPi (green, *n* = 7), [^18^F]FPyPARP (red, *n* = 7), and [^18^F]FTT (blue, *n* = 6) in comparison. **B** Tumor-to-muscle, tumor-to-blood, and liver-to-kidney ratios 1.5 h (lighter colors) and 2.5 h p.i. (darker colors). **C** PARP1 immunofluorescence microscopy images. PARP1 is displayed in red and nuclei in green and the scale bar represents 20 µm

A standardized study protocol was applied for all in vivo experiments where immunodeficient mice were injected with HCC1937 cells subcutaneously. After the xenografts reached a sufficient size (302 ± 152 mm^3^), the respective tracer was injected i.v. and dynamic as well as static PET data and anatomical MR images were acquired and biodistribution was analyzed ex vivo using gamma-counting. Since specificity of the uptake was already demonstrated in the original publications of the gold standard tracers [22, 24] and (in this study) in vitro, in vivo blocking controls were not considered necessary. PET images showed high excretion-related abdominal signal throughout all groups but interestingly, for [^18^F]FTT, no noticeable renal clearance was observed as indicated by the absence of bladder uptake in contrast to [^18^F]PARPi, where some of the animals showed renal excretion and [^18^F]FPyPARP with all animals exhibiting strong radioactive signal in the bladder (Fig. 2C, Supplementary Figs. 6 and 7). Radiotracer uptake was found to be heterogeneously distributed within the xenografts which is in line with the general heterogeneity of PARP1 expression in xenografts [35, 36] and confirmed by ex vivo immunofluorescence microscopy (Fig. 3C). The ex vivo biodistribution analyses at 1.5 h and 2.5 h were comparable in their absolute organ uptake values within the cohorts and confirmed high abdominal uptake especially in the liver, intestine, and kidney (Fig. 2D, E). The TACs from the dynamically acquired PET data revealed a higher overall xenograft uptake for [^18^F]FTT whereas [^18^F]FPyPARP and [^18^F]PARPi were within the same range. As already indicated by the bladder uptake pointing towards partial renal clearance, the liver TACs revealed lower uptake values in the [^18^F]FPyPARP cohort in direct comparison to [^18^F]PARPi at early time points (Fig. 3A, Supplementary Fig. 8). The tumor uptake ratio showed significantly lower values for [^18^F]FPyPARP compared to [^18^F]PARPi at 1.5 h p.i. (mean values of 1.99 and 5.3, respectively, *p* = 0.0098) and compared to [^18^F]FTT at 2.5 h p.i. (mean values of 2.46 and 3.42, respectively, *p* = 0.0182) relative to muscle tissue as control (tumor-to-muscle ratio, TMR). If referenced to blood uptake (tumor-to-blood ratio, TBR), the ratios of the [^18^F]FPyPARP cohort are higher after 2.5 h p.i. (*p* = 0.0649, ns) compared to [^18^F]FTT (mean values of 3.99 and 2.46, respectively, Fig. 3B). To determine the potential for imaging of liver metastasis, the tumor-to-liver ratios (TLR) were compared. Although the TLR of [^18^F]FTT was significantly higher at both time points, all TLRs exhibit a value lower than 1 (Supplementary Fig. 9). For comparison of the excretion routes, the liver-to-kidney ratio (LKR) was calculated and found to be significantly lower for the [^18^F]FPyPARP cohort compared to the [^18^F]PARPi cohort, although only at the 2.5-h time point (mean values of 0.60 and 1.37, respectively, *p* = 0.0224). Autoradiographs 2.5 h post-injection show even hepatic signal for all tracers and pronounced cortical renal signal for [^18^F]PARPi, while both other tracers are characterized by more medullar uptake (Supplementary Fig. 6B); however since this only represents one time point and we observed inconsistent spacing to the radiography screen in our experimental setup and thus suboptimal resolution, these results should be taken with caution.

## Discussion

The current PARP radiotracers are rapidly taken up into the liver and cleared via bile in rodents and are thus suboptimal for imaging of abdominal lesions. In order to identify the PARP radiotracer most suitable for this purpose, we aimed to compare the most relevant tracers in the same animal model. In addition, we synthesized a variant with reduced lipophilicity with the intention to shift the excretion route towards renal clearance. This was based on the observation that the physicochemical parameter “lipophilicity” is apparently a good parameter for many drugs to predict their excretion route [33].

We automated radiosynthesis procedures for the two literature-known probes and our novel PARP tracer in order to compare them side-by-side in regard to clearance route and tumor uptake. While [^18^F]FTT radiosynthesis only requires one step, [^18^F]PARPi synthesis developed by Carney et al. [22] involves many additions of low reagent volumes and has not been automated in literature. We instead successfully used our established automated [^18^F]SFB labeling protocol for synthesis of this compound although during the preparation of the manuscript, a one-pot synthesis of [^18^F]PARPi has been published by Wilson et al. [37]. To synthesize the novel variant [^18^F]FPyPARP, we designed a one-pot reaction utilizing the synthon [^18^F]FPyTFP. This synthesis can be further simplified in future by late-stage labeling of an already conjugated trimethylammonium nicotinate precursor. The radiotracer is highly stable in mouse as well as human serum; however, first-in-human clinical studies of [^18^F]PARPi showed a radiometabolite as early as 25 min [32] which presumably can be attributed to tissue metabolism. Since [^18^F]FPyPARP and [^18^F]PARPi are structurally similar, it is likely that their metabolism follows the same pathway. Experimentally determined logD values, which are more relevant for in vivo studies as they take the physiological pH into account, confirmed the reduced lipophilicity of [^18^F]FPyPARP in comparison to [^18^F]PARPi and [^18^F]FTT by a factor of 10.

Of note, PARP inhibitors need to enter the cells to reach their intranuclear targets. The radiotracer uptake experiments clearly demonstrated high cell-associated uptake in the chosen HCC1937 cells that was blockable to baseline by olaparib, indicating specific interactions of all three tracers with their intranuclear targets. This was further confirmed by the comparison of HCC1937 washed with either medium or an acidic glycine buffer that did not show any reduction in the radiotracer signal that would hint to ionic binding to the cell surface. The mechanisms of cellular uptake of PARP inhibitor across the membrane are currently unknown. Because of the well-known role of membrane transporters in drug uptake [38–40], we speculate that the three radiotracers are also substrates of solute carrier (SLC) uptake transporters. An in silico analysis of transporter gene expression in HCC1937 cells identified a number of expressed SLC transporters (Supplementary Table 2). Within the SLC families implicated in drug transport, several SLC transporters might be candidates of PARP inhibitor uptake (e.g., SLC22A5, SLC22A18, SLC29A1, SLC29A2, SLCO3A1, SLCO4A1). In-depth functional characterization of these transporter candidates warrants further investigation, which is beyond the scope of this article. Of interest, ABCB1 (encoding MDR1 P-glycoprotein), which had been identified as efflux transporter of olaparib [41], is not expressed in HCC1937 cells.

The in vivo analyses employed a standardized protocol to ensure comparability within the different animal cohorts and indeed revealed decent xenograft uptake. Despite the lower TMRs of the [^18^F]FPyPARP cohort caused by higher muscle uptake of this group, the TBRs indicate more effective blood clearance in comparison to [^18^F]FTT and thus slightly lower unspecific background, while on a similar level with [^18^F]PARPi. The TLRs below 1 suggest that all three radiotracers are not optimal for the imaging of abdominal lesions in mice. Calculated blood half-life was highest for [^18^F]PARPi in comparison to [^18^F]FPyPARP and [^18^F]FTT (3 ± 0.7 min, 2.6 ± 1.4 min and 2.1 ± 0.5 min, respectively); nevertheless, the blood activity of [^18^F]FTT remains the highest of the three at early time points (Fig. 3A). The preference of either radiotracer is thus dependent on the choice of reference tissue.

All radiotracers showed high abdominal uptake particularly in the liver, spleen, kidneys, and intestines. These findings point towards mainly hepatobiliary clearance of all three radiotracers; however, only for [^18^F]FPyPARP bladder uptake was detected in all animals indicating an elevated level of renal clearance in contrast to [^18^F]PARPi and [^18^F]FTT. This is additionally supported by a detailed look at the TACs showing lower initial liver uptake of [^18^F]FPyPARP compared to [^18^F]PARPi. Furthermore, liver-to-kidney ratios decreased between the 1.5 and 2.5 h time points only for [^18^F]FPyPARP, while no significant decrease was observed for the other tracers (Fig. 3B, right graph). The decrease in this group was caused by a lower retention of the signal in the liver compared to kidney tissue. Thus, the reduced logD of our novel tracer resulted in higher renal than hepatobiliary excretion, although only to a minor degree. The recently published [^18^F]olaparib only shows hepatobiliary clearance although it has an even lower calculated logP value of 2.02 [42] than [^18^F]FPyPARP or [^18^F]PARPi. The reported logD value of olaparib, however, is higher than the experimentally determined logD of [^18^F]FPyPARP (Lynparza monograph, AstraZeneca: 1.49, vs 1.16, respectively), suggesting that the logD is a more accurate parameter than logP for the prediction of clearance routes. This underlines that the physicochemical parameter “lipophilicity” may be a good predictor for the clearance pathways of many drugs, yet it does not account for all the different processes involved in hepatobiliary and renal clearance. Mechanisms such as transporter-mediated cellular uptake and efflux are important determinants of drug distribution and excretion [38–40]. The involvement of membrane transporters in the cellular uptake of PARPi radiotracers, beyond the role of ABCB1 in cellular efflux of olaparib [41], is currently unknown and will be studied in further investigations.

## Conclusion

We here present the alternative PARP imaging agent [^18^F]FPyPARP and compare it to the benchmark tracers [^18^F]PARPi and [^18^F]FTT in the same in vivo model. In contrast to its parent compound, [^18^F]PARPi, [^18^F]FPyPARP features both a facilitated synthesis route and reduced lipophilicity. Side-by-side comparison of the three radiotracers revealed tumor-to-tissue ratios in the same range although minor differences were observed depending on the reference tissue. However, [^18^F]FPyPARP was the only radiotracer showing a significant decrease in the LKR when comparing early to late time points, which hints towards a lower retention of this molecule in liver tissue compared to the benchmark radiotracers. This is of particular interest since low retention in non-target tissue is beneficial for targeted radiotherapy of PARP overexpressing lesions that bears high potential of a targeted therapy with promising first outcomes [43–45].

According to the obtained PET/MR images and the resulting TACs, [^18^F]FPyPARP excretion is partially renal, demonstrating that small changes in the molecule can have a beneficial influence on the pharmacokinetics without affecting the uptake performance. Our data highlight the advantages of the three different radiotracers: [^18^F]PARPi exhibits the highest initial TMR, [^18^F]FPyPARP demonstrated improved clearance from liver tissue and sufficient tumor uptake, and [^18^F]FTT showed continuously increasing tumor uptake due to the long blood retention time. This is in line with recent findings on the different modes of action of various PARP inhibitors, warranting the exploration of different pharmacophores for imaging to address unmet needs [46].

Since clinical data obtained by the group of Thomas Reiner indicate that [^18^F]PARPi already has a 30% renal clearance in humans [32], it can be concluded that our radiotracer might have even lower excretion-related abdominal background signal in humans. With these data, [^18^F]FPyPARP has emerged as decent radiotracer for PARP expression with the benefit of improved renal clearance.

## Supplementary Information

Below is the link to the electronic supplementary material.

Supplementary file1 (DOCX 2765 KB)

## Data Availability

Primary data are available upon reasonable request.

## References

[1] Morales J, Li L, Fattah FJ, Dong Y, Bey EA, Patel M (2014). Review of poly (ADP-ribose) polymerase (PARP) mechanisms of action and rationale for targeting in cancer and other diseases. Crit Rev Eukaryot Gene Expr.

[2] Javle M, Curtin NJ (2011). The role of PARP in DNA repair and its therapeutic exploitation. Br J Cancer.

[3] Kyle S, Thomas HD, Mitchell J, Curtin NJ. Exploiting the Achilles heel of cancer: the therapeutic potential of poly(ADP-ribose) polymerase inhibitors in BRCA2-defective cancer. Br J Radiol. 2008;81 Spec No 1:S6–11. doi:10.1259/bjr/99111297. 10.1259/bjr/99111297 18820000

[4] Audeh MW, Penson RT, Friedlander M, Powell B, Bell-McGuinn KM, Scott C et al. Phase II trial of the oral PARP inhibitor olaparib (AZD2281) in BRCA-deficient advanced ovarian cancer. Journal of Clinical Oncology. 2009;27(15_suppl):5500-. doi:10.1200/jco.2009.27.15_suppl.5500.

[5] Lord CJ, Tutt AN, Ashworth A (2015). Synthetic lethality and cancer therapy: lessons learned from the development of PARP inhibitors. Annu Rev Med.

[6] Shen Y, Rehman FL, Feng Y, Boshuizen J, Bajrami I, Elliott R (2013). BMN 673, a novel and highly potent PARP1/2 inhibitor for the treatment of human cancers with DNA repair deficiency. Clin Cancer Res.

[7] Lord CJ, Ashworth A (2017). PARP inhibitors: Synthetic lethality in the clinic. Science.

[8] Pommier Y, O'Connor MJ, de Bono J. Laying a trap to kill cancer cells: PARP inhibitors and their mechanisms of action. Sci Transl Med. 2016;8(362):362ps17. doi:10.1126/scitranslmed.aaf9246. 10.1126/scitranslmed.aaf9246 27797957

[9] Helleday T (2011). The underlying mechanism for the PARP and BRCA synthetic lethality: clearing up the misunderstandings. Mol Oncol.

[10] Maya-Mendoza A, Moudry P, Merchut-Maya JM, Lee M, Strauss R, Bartek J (2018). High speed of fork progression induces DNA replication stress and genomic instability. Nature.

[11] Ellisen LW (2011). PARP inhibitors in cancer therapy: promise, progress, and puzzles. Cancer Cell.

[12] Domchek SM, Aghajanian C, Shapira-Frommer R, Schmutzler RK, Audeh MW, Friedlander M (2016). Efficacy and safety of olaparib monotherapy in germline BRCA1/2 mutation carriers with advanced ovarian cancer and three or more lines of prior therapy. Gynecol Oncol.

[13] Ledermann J, Harter P, Gourley C, Friedlander M, Vergote I, Rustin G (2012). Olaparib maintenance therapy in platinum-sensitive relapsed ovarian cancer. N Engl J Med.

[14] Pilie PG, Gay CM, Byers LA, O'Connor MJ, Yap TA (2019). PARP Inhibitors: Extending Benefit Beyond BRCA-Mutant Cancers. Clin Cancer Res.

[15] Li X, Li C, Jin J, Wang J, Huang J, Ma Z (2018). High PARP-1 expression predicts poor survival in acute myeloid leukemia and PARP-1 inhibitor and SAHA-bendamustine hybrid inhibitor combination treatment synergistically enhances anti-tumor effects. EBioMedicine.

[16] Siraj AK, Pratheeshkumar P, Parvathareddy SK, Divya SP, Al-Dayel F, Tulbah A (2018). Overexpression of PARP is an independent prognostic marker for poor survival in Middle Eastern breast cancer and its inhibition can be enhanced with embelin co-treatment. Oncotarget.

[17] Ossovskaya V, Koo IC, Kaldjian EP, Alvares C, Sherman BM (2010). Upregulation of Poly (ADP-Ribose) Polymerase-1 (PARP1) in Triple-Negative Breast Cancer and Other Primary Human Tumor Types. Genes Cancer.

[18] Carney B, Kossatz S, Reiner T (2017). Molecular Imaging of PARP. J Nucl Med.

[19] Reiner T, Keliher EJ, Earley S, Marinelli B, Weissleder R (2011). Synthesis and in vivo imaging of a 18F-labeled PARP1 inhibitor using a chemically orthogonal scavenger-assisted high-performance method. Angew Chem Int Ed Engl.

[20] Reiner T, Lacy J, Keliher EJ, Yang KS, Ullal A, Kohler RH (2012). Imaging therapeutic PARP inhibition in vivo through bioorthogonally developed companion imaging agents. Neoplasia.

[21] Zhou D, Xu J, Mpoy C, Chu W, Kim SH, Li H (2018). Preliminary evaluation of a novel (18)F-labeled PARP-1 ligand for PET imaging of PARP-1 expression in prostate cancer. Nucl Med Biol.

[22] Carney B, Carlucci G, Salinas B, Di Gialleonardo V, Kossatz S, Vansteene A (2016). Non-invasive PET Imaging of PARP1 Expression in Glioblastoma Models. Mol Imaging Biol.

[23] Zhou D, Chu W, Xu J, Jones LA, Peng X, Li S (2014). Synthesis, [(1)(8)F] radiolabeling, and evaluation of poly (ADP-ribose) polymerase-1 (PARP-1) inhibitors for in vivo imaging of PARP-1 using positron emission tomography. Bioorg Med Chem.

[24] Edmonds CE, Makvandi M, Lieberman BP, Xu K, Zeng C, Li S (2016). [(18)F]FluorThanatrace uptake as a marker of PARP1 expression and activity in breast cancer. Am J Nucl Med Mol Imaging.

[25] Carney B, Kossatz S, Lok BH, Schneeberger V, Gangangari KK, Pillarsetty NVK (2018). Target engagement imaging of PARP inhibitors in small-cell lung cancer. Nat Commun.

[26] Makvandi M, Pantel A, Schwartz L, Schubert E, Xu K, Hsieh CJ (2018). A PET imaging agent for evaluating PARP-1 expression in ovarian cancer. J Clin Invest.

[27] Sander Effron S, Makvandi M, Lin L, Xu K, Li S, Lee H (2017). PARP-1 Expression Quantified by [(18)F]FluorThanatrace: A Biomarker of Response to PARP Inhibition Adjuvant to Radiation Therapy. Cancer Biother Radiopharm.

[28] Kossatz S, Brand C, Gutiontov S, Liu JT, Lee NY, Gonen M (2016). Detection and delineation of oral cancer with a PARP1 targeted optical imaging agent. Sci Rep.

[29] Kossatz S, Pirovano G, De Souza França PD, Strome AL, Sunny SP, Zanoni DK et al. PARP1 as a biomarker for early detection and intraoperative tumor delineation in epithelial cancers – first-in-human results. bioRxiv. 2019:663385. doi:10.1101/663385.

[30] Carlucci G, Carney B, Brand C, Kossatz S, Irwin CP, Carlin SD (2015). Dual-Modality Optical/PET Imaging of PARP1 in Glioblastoma. Mol Imaging Biol.

[31] Lin LL. Serial Imaging of the Novel Radiotracer ^18F FLuorthanatrace ( ^18F FTT) by PET/CTF. 2018. https://ClinicalTrials.gov/show/NCT03604315.

[32] Schoder H, Franca PDS, Nakajima R, Burnazi E, Roberts S, Brand C (2020). Safety and Feasibility of PARP1/2 Imaging with (18)F-PARPi in Patients with Head and Neck Cancer. Clin Cancer Res.

[33] Wakayama N, Toshimoto K, Maeda K, Hotta S, Ishida T, Akiyama Y (2018). In Silico Prediction of Major Clearance Pathways of Drugs among 9 Routes with Two-Step Support Vector Machines. Pharm Res.

[34] Olberg DE, Arukwe JM, Grace D, Hjelstuen OK, Solbakken M, Kindberg GM (2010). One step radiosynthesis of 6-[(18)F]fluoronicotinic acid 2,3,5,6-tetrafluorophenyl ester ([(18)F]F-Py-TFP): a new prosthetic group for efficient labeling of biomolecules with fluorine-18. J Med Chem.

[35] Bertucci F, Finetti P, Monneur A, Perrot D, Chevreau C, Le Cesne A (2019). PARP1 expression in soft tissue sarcomas is a poor-prognosis factor and a new potential therapeutic target. Mol Oncol.

[36] Zmuda F, Blair A, Liuzzi MC, Malviya G, Chalmers AJ, Lewis D (2018). An (18)F-Labeled Poly(ADP-ribose) Polymerase Positron Emission Tomography Imaging Agent. J Med Chem.

[37] Wilson TC, Pillarsetty N, Reiner T (2020). A one-pot radiosynthesis of [(18) F]PARPi. J Labelled Comp Radiopharm.

[38] Giacomini KM, Huang SM, Tweedie DJ, Benet LZ, Brouwer KL, International Transporter C (2010). Membrane transporters in drug development. Nat Rev Drug Discov.

[39] Nies AT, Niemi M, Burk O, Winter S, Zanger UM, Stieger B (2013). Genetics is a major determinant of expression of the human hepatic uptake transporter OATP1B1, but not of OATP1B3 and OATP2B1. Genome Med.

[40] Neul C, Schaeffeler E, Sparreboom A, Laufer S, Schwab M, Nies AT (2016). Impact of Membrane Drug Transporters on Resistance to Small-Molecule Tyrosine Kinase Inhibitors. Trends Pharmacol Sci.

[41] Lawlor D, Martin P, Busschots S, Thery J, O'Leary JJ, Hennessy BT (2014). PARP Inhibitors as P-glyoprotein Substrates. J Pharm Sci.

[42] Wilson TC, Xavier MA, Knight J, Verhoog S, Torres JB, Mosley M (2019). PET Imaging of PARP Expression Using (18)F-Olaparib. J Nucl Med.

[43] Jannetti SA, Carlucci G, Carney B, Kossatz S, Shenker L, Carter LM (2018). PARP-1-Targeted Radiotherapy in Mouse Models of Glioblastoma. J Nucl Med.

[44] Pirovano G, Jannetti SA, Carter LM, Sadique A, Kossatz S, Guru N (2020). Targeted brain tumor radiotherapy using an Auger emitter. Clin Cancer Res.

[45] Salinas B, Irwin CP, Kossatz S, Bolaender A, Chiosis G, Pillarsetty N (2015). Radioiodinated PARP1 tracers for glioblastoma imaging. EJNMMI Res.

[46] Zandarashvili L, Langelier MF, Velagapudi UK, Hancock MA, Steffen JD, Billur R et al. Structural basis for allosteric PARP-1 retention on DNA breaks. Science. 2020;368(6486). doi:10.1126/science.aax6367. 10.1126/science.aax6367 PMC7347020 32241924

