# Supplementary material for: Two experts and a newbie: [18F]PARPi vs [18F]FTT vs [18F]FPyPARP—a comparison of PARP imaging agents
Source: Eur J Nucl Med Mol Imaging. 2021 Sep 6;49(3):834–46. doi: 10.1007/s00259-021-05436-7 (PMC8803746; doi:10.1007/s00259-021-05436-7)
Supplement: Supplementary file 1 — Supplementary file1 (DOCX 2765 KB) [file 259_2021_5436_MOESM1_ESM.docx]

**Supplementary Information**

**for**

**Two Experts and a Newbie: [^18^F]PARPi vs [^18^F]FTT vs [^18^F]FPyPARP – A Comparison of PARP Imaging Agents.**

Submitted to the European Journal of Nuclear Medicine and Molecular Imaging

Sophie Stotz^1,2^, Johannes Kinzler^1^, Anne T. Nies^2,3^, Matthias Schwab^2,3,4^, and Andreas Maurer^1,2^

^1^ Werner Siemens Imaging Center, Department of Preclinical Imaging and Radiopharmacy, Eberhard Karls University, Tuebingen, Germany

^2^ Cluster of Excellence iFIT (EXC 2180) "Image Guided and Functionally Instructed Tumor Therapies", Eberhard Karls University, Tuebingen, Germany

^3^ Dr. Margarete Fischer-Bosch-Institute of Clinical Pharmacology, Stuttgart, and University of Tuebingen, Tuebingen, Germany

^4^ Departments of Clinical Pharmacology, and of Biochemistry and Pharmacy, University of Tuebingen, Tuebingen, Germany

**Corresponding Author**

Dr. rer. nat. Andreas Maurer (ORCID: 0000-0003-2412-5361)

Email andr3asmaur3r@gmail.com


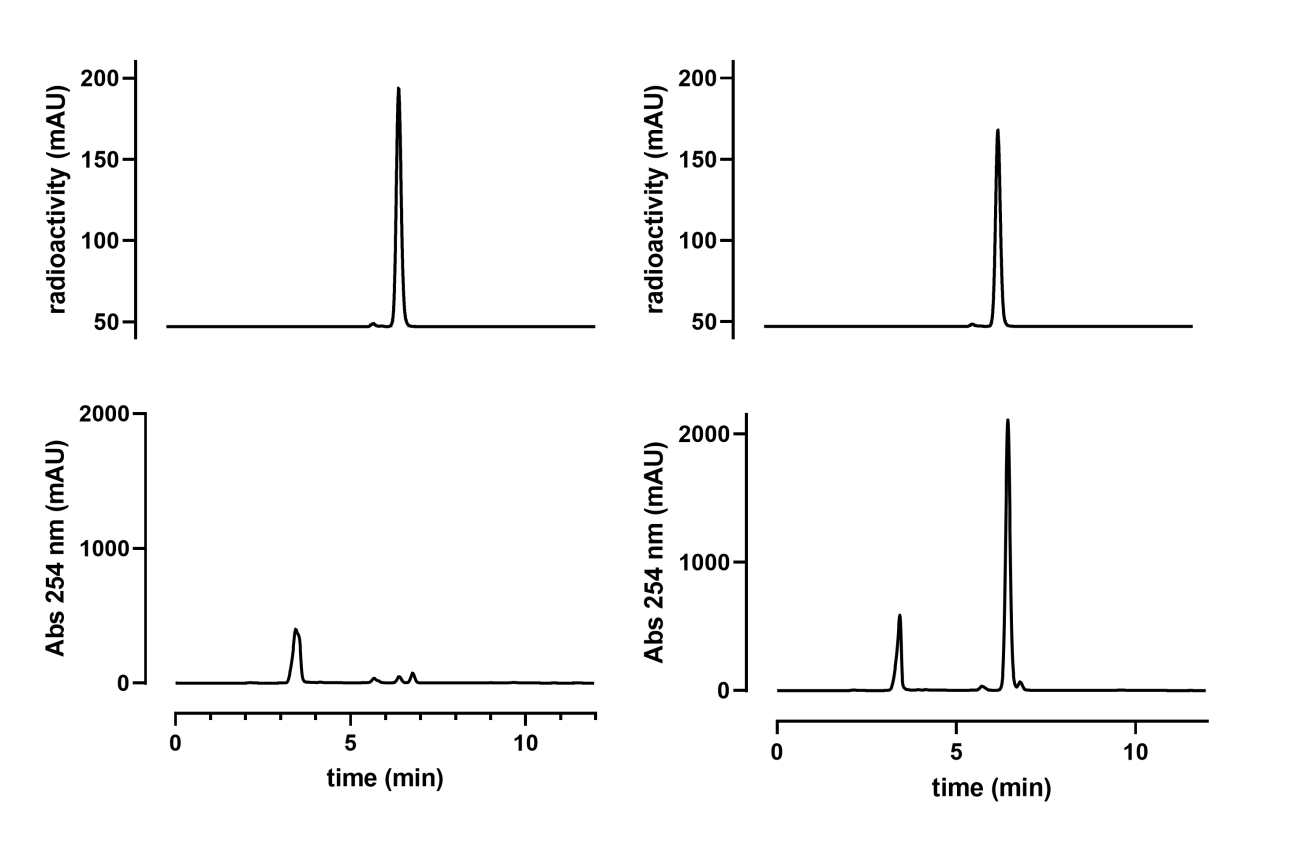


Supplementary Figure 1: Exemplary HPLC analysis of [^18^F]PARPi by spiking with the nonradioactive PARPi. Radioactivity (upper signal) and UV absorbance chromatograms before addition of the non-radioactive PARPi (left side) and of spiked samples revealing an UV peak at the same retention time as the radioactivity peak (8 min, right side). The UV peak at approximately 4 min is caused by residual acetone from the module cleaning.


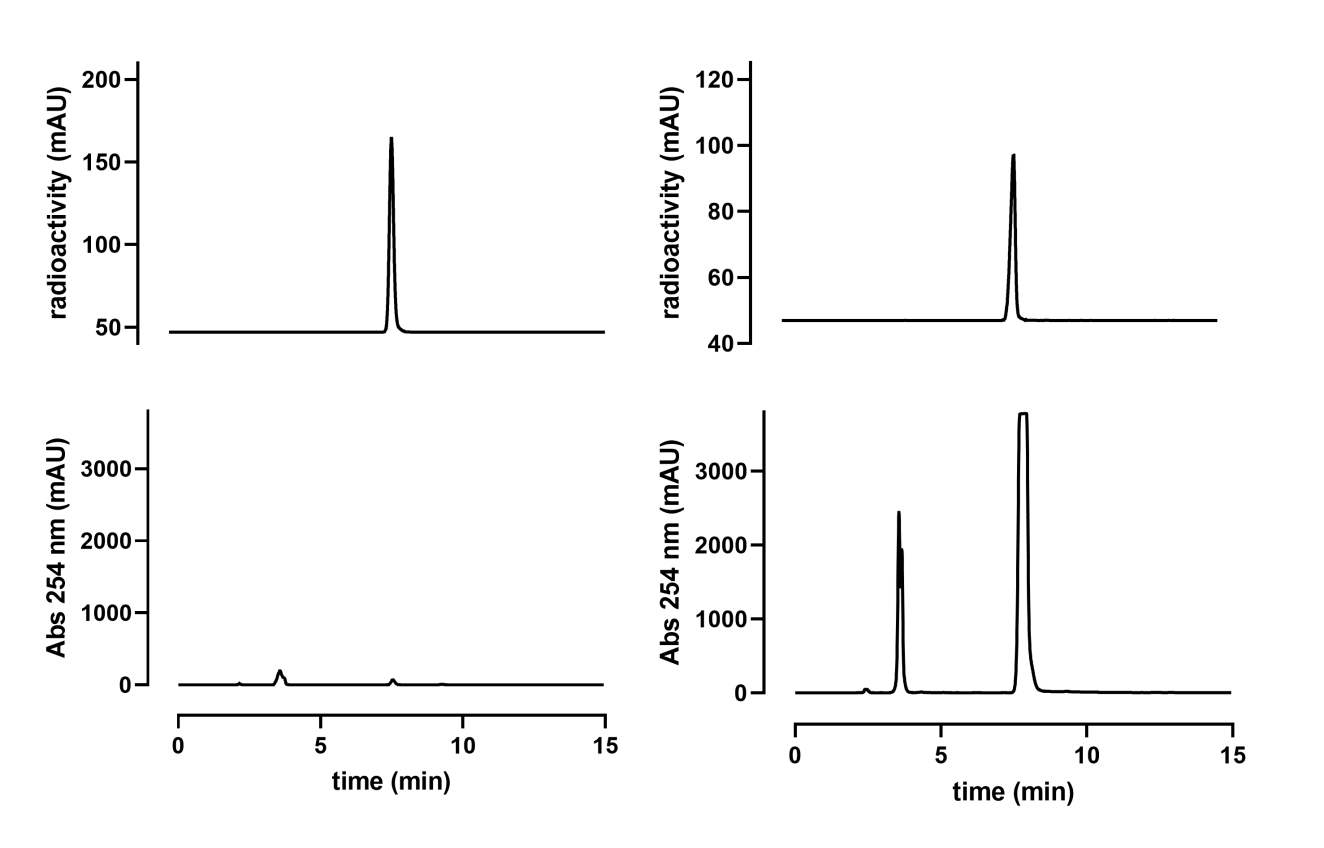


Supplementary Figure 2: Exemplary HPLC analysis of [^18^F]FPyPARP by spiking with the nonradioactive FPyPARP. Radioactivity (upper signal) and UV absorbance chromatograms before addition of the non-radioactive FPyPARP (left side) and of spiked samples revealing an UV peak at the same retention time as the radioactivity peak (9-10 min, right side). The UV peak at approximately 4 min is caused by residual acetone.


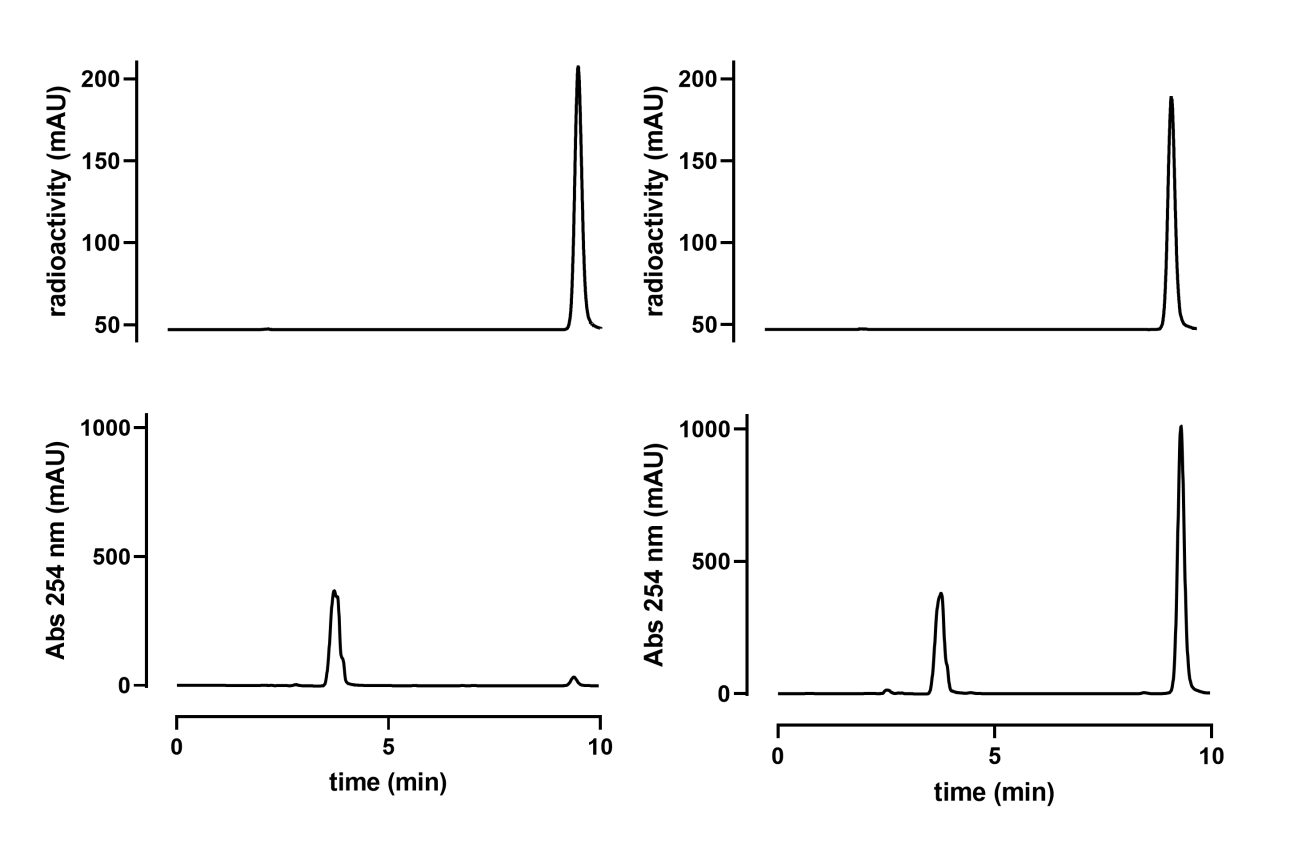


Supplementary Figure 3: Exemplary HPLC analysis of [^18^F]FTT by spiking with nonradioactive FTT. Radioactivity (upper signal) and UV absorbance chromatograms before addition of the non-radioactive FTT (left side) and of spiked samples revealing an UV peak at the same retention time as the radioactivity peak (6-7 min, right side). The UV peak at approximately 4 min is caused by residual acetone.


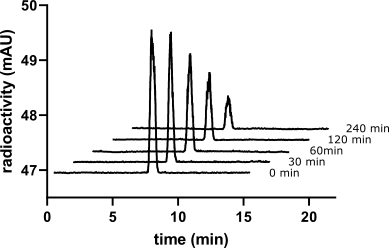


Supplementary Figure 4: Human serum stability analysis. Samples of human serum mixed with [^18^F]FPyPARP were drawn after 0, 30, 60, 120 and 240 min.


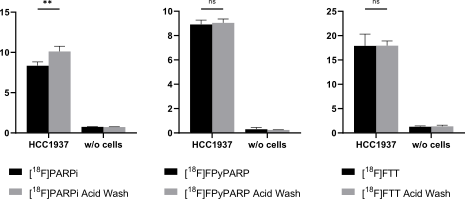


Supplementary Figure 5: Uptake of the radiotracers in HCC1937 cells washed with medium compared to uptake in HCC1937 cells washed with an acidic glycine buffer. No difference was observed for [^18^F]FPyPARP and [^18^F]FTT while [^18^F]PARPi showed a significantly higher uptake in cells washed with the acidic buffer (p = 0.0087).


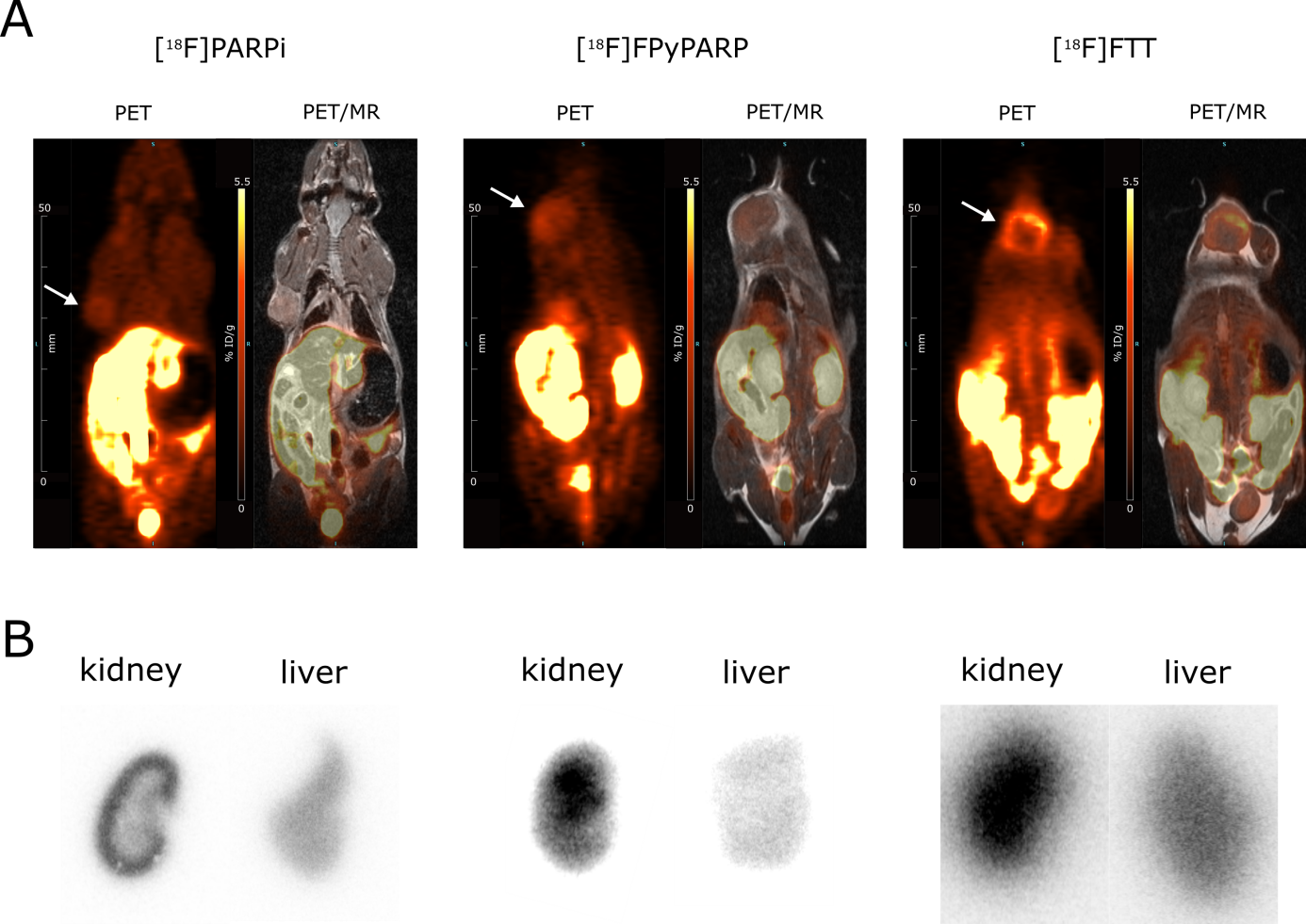


Supplementary Figure 6: Representative images of static PET and MR scans 2 h p.i. and autoradiographs of liver and kidney sections. A Representative PET/MR images of mice injected with either [^18^F]PARPi, [^18^F]FPyPARP and [^18^F]FTT. Tumors are indicated with white arrows. B Representative autoradiography images of kidney and liver sections of one animal each. The apparent differences in resolution are caused by uneven spacing of the different samples to the screen within the cassette.


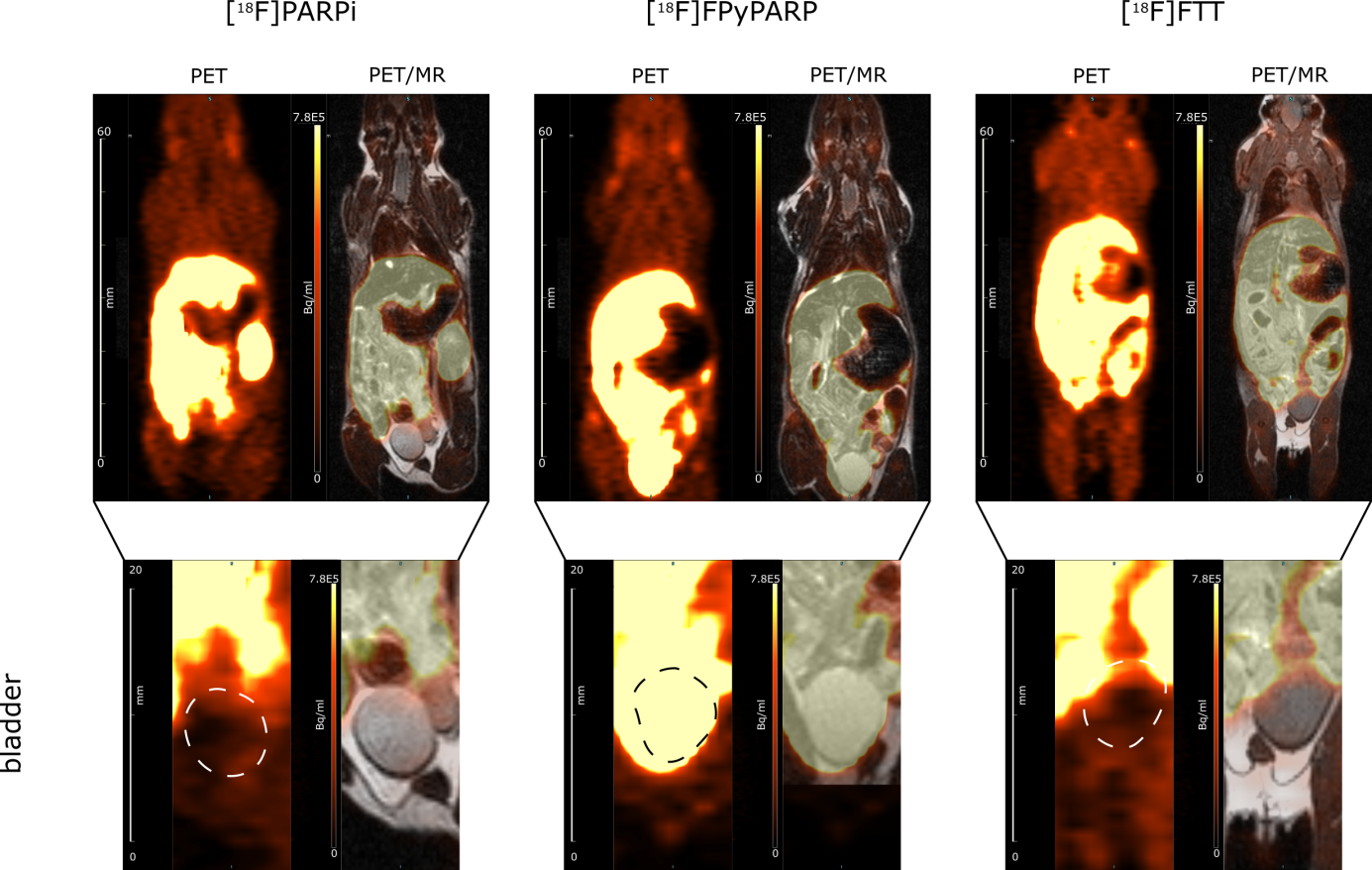


Supplementary Figure 7: Representative PET and close-up images of bladders below at the last 10 min of a 1 h dynamic PET scan. Size bars represent 60 mm (whole body) or 20 mm (bladders) and color-coded intensity bars range from 0 to 7.8×10^5^ Bq/ml. Bladders are marked in white ([^18^F]PARPi, [^18^F]FTT) or black ([^18^F]FPyPARP).


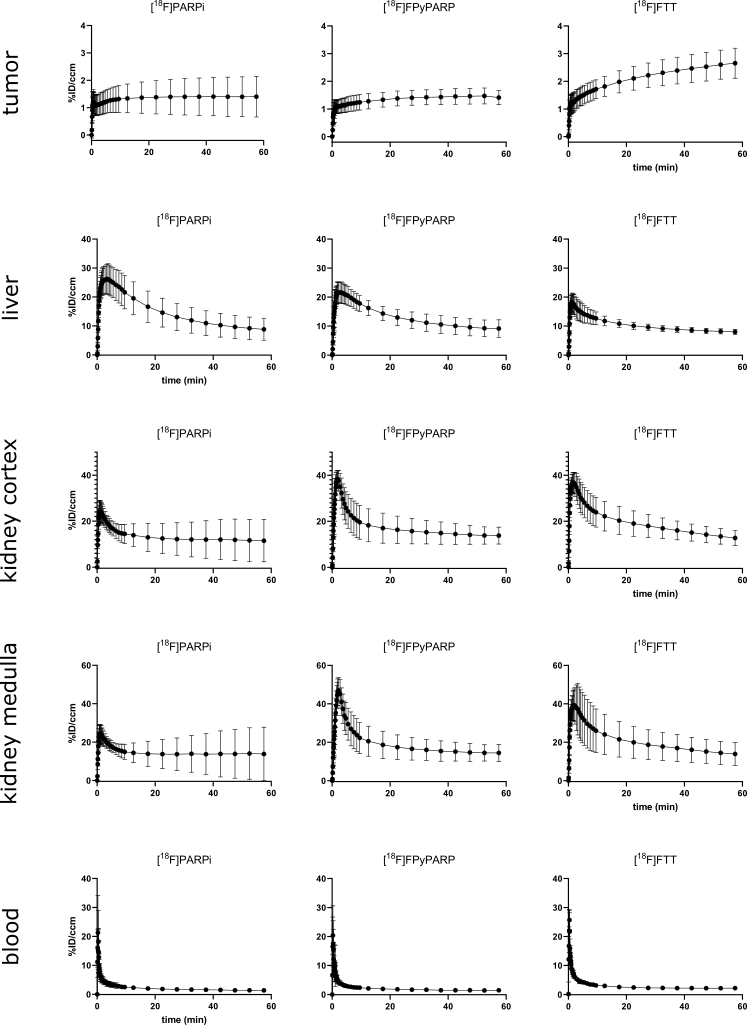


Supplementary Figure 8: Time-activity-curves displayed by respective radiotracer. TACs of liver, kidney (separated in medulla and cortex), tumor and heart of [^18^F]PARPi, [^18^F]FPyPARP and [^18^F]FTT in comparison.


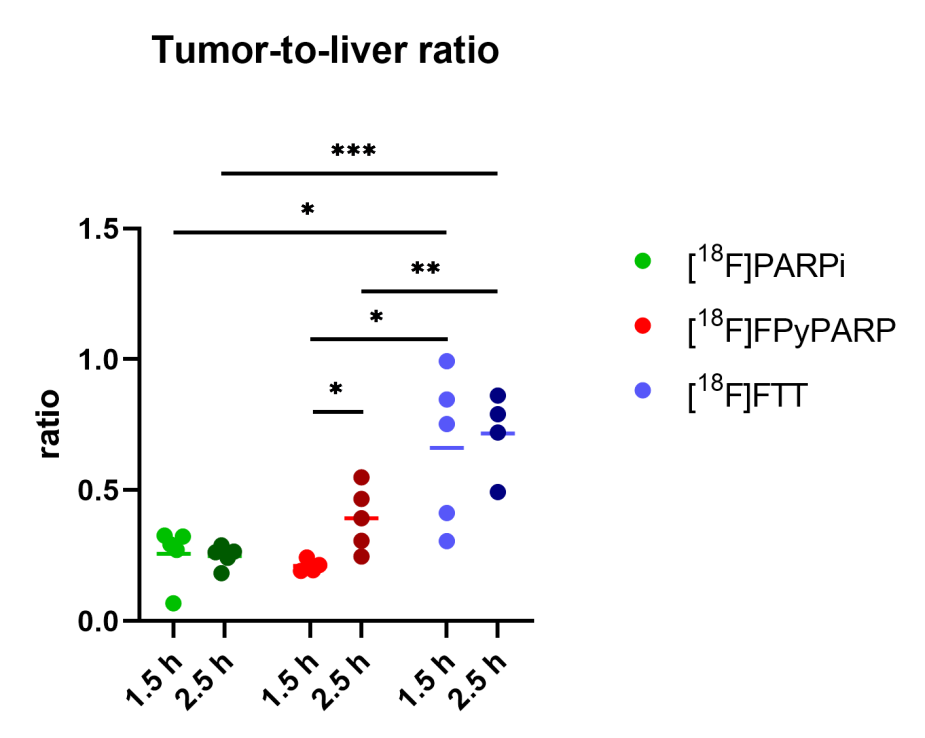


Supplementary Figure 9: Comparison of the tumor-to-liver ratios of the three radiotracers. [^18^F]FTT shows significantly higher ratios at both time points compared to [^18^F]PARPi and [^18^F]FPyPARP. The TLR of [^18^F]FPyPARP is significantly higher after 2.5 h compared to 1.5 h (p = 0.0225).

**Supplementary Table 1: Additional radiotracer synthesis details for [^18^F]PARPi, [^18^F]FPyPARP and [^18^F]FTT.**

|  | **Synthesis No** | **Start activity (GBq)** | **Product activity (GBq)** | **Synthesis time (min)** | **DCY (%)** | **Specific activity (GBq/µmol)** |
| --- | --- | --- | --- | --- | --- | --- |
| **[^18^F]PARPi** | 1 | 51 | 2.6 | 95 | 9.3 | 51.92 |
|  | 2 | 46 | 3.6 | 98 | 14.5 | 51.14 |
|  | 3 | 33 | 1.4 | 106 | 8.1 | 68.45 |
|  | 4 | 45 | 1.9 | 97 | 7.9 | 63.7 |
|  | 5 | 52 | 1.9 | 105 | 7.1 | 47.48 |
|  | 6 | 55 | 2.4 | 93 | 7.9 | 60.1 |
|  | 7 | 36 | 1.2 | 106 | 6.4 | 38.97 |
|  | 8 | 47 | 1.3 | 91 | 4.8 | 31.45 |
|  | 9 | 30 | 0.9 | 92 | 5.4 | 47.08 |
|  | 10 | 56 | 1.8 | 92 | 5.6 | 116.31 |
|  | 11 | 55 | 0.2 | 90 | 0.7 | 65.55 |
|  | 12 | 62 | 1.5 | 91 | 4.4 | 265.18 |
|  | 13 | 57 | 1.33 | 88 | 4.0 | 166.6 |
|  | 14 | 63 | 1.0 | 91 | 2.9 | 60.76 |
|  | 15 | 64 | 2.0 | 87 | 5.5 | 52.76 |
|  | 16 | N/A | 4.7 | N/A | N/A | 87.39 |
|  | 17 | 46 | 3.0 | 89 | 11.5 | 67.98 |
| **[^18^F]FPyPARP** | 1 | 16.5 | 1.0 | 74 | 9.4 | 16.16 |
|  | 2 | 31 | 3.2 | 74 | 16.3 | 24.92 |
|  | 3 | 52.5 | 6.5 | 71 | 19.6 | 32.28 |
|  | 4 | 44 | 0.3 | 63 | 1.1 | 18.81 |
|  | 5 | 35.5 | 2 | 73 | 9.0 | 33.46 |
|  | 6 | 75 | 8.2 | 76 | 17.7 | 57.46 |
|  | 7 | 38 | 1.2 | 69 | 4.7 | 33.72 |
|  | 8 | 41 | 0.5 | 68 | 1.8 | 28.20 |
| **[^18^F]FTT** | 1 | 27 | 1.2 | 52 | 6.2 | 71.18 |
|  | 2 | 52 | 2.5 | 55 | 6.7 | 130.70 |
|  | 3 | 52 | 3.3 | 51 | 8.8 | 178.00 |
|  | 4 | 57 | 6.8 | 48 | 16.3 | 135.87 |

**Supplementary Table 2: Transporter expression in HCC1937 cells.** In silico analysis of transporter gene expression in HCC1937 cells of the Cancer Cell Line Encyclopedia (CCLE) [1]. RSEM RNAseq gene expression data were downloaded from the DepMap portal (https://depmap.org/portal/ccle/) and all SLC (solute carrier) and ABC (ATP-binding cassette) gene probe sets with an NM_ accession prefix were extracted yielding 385 SLC and 48 ABC genes. The SLC17, SLC18, SLC25, SLC32 and SLC35 family are not shown because they encode intracellularly-localized transporters [2]. Families SLC22, SLC28, SLC29, SLC47 and SLCO are highlighted because they have been implicated in drug transport [3–8]. Also, the selected ABC transporters have been implicated in drug transport [9, 10]. Expression data of PARP1 and PARP2 are shown as well.


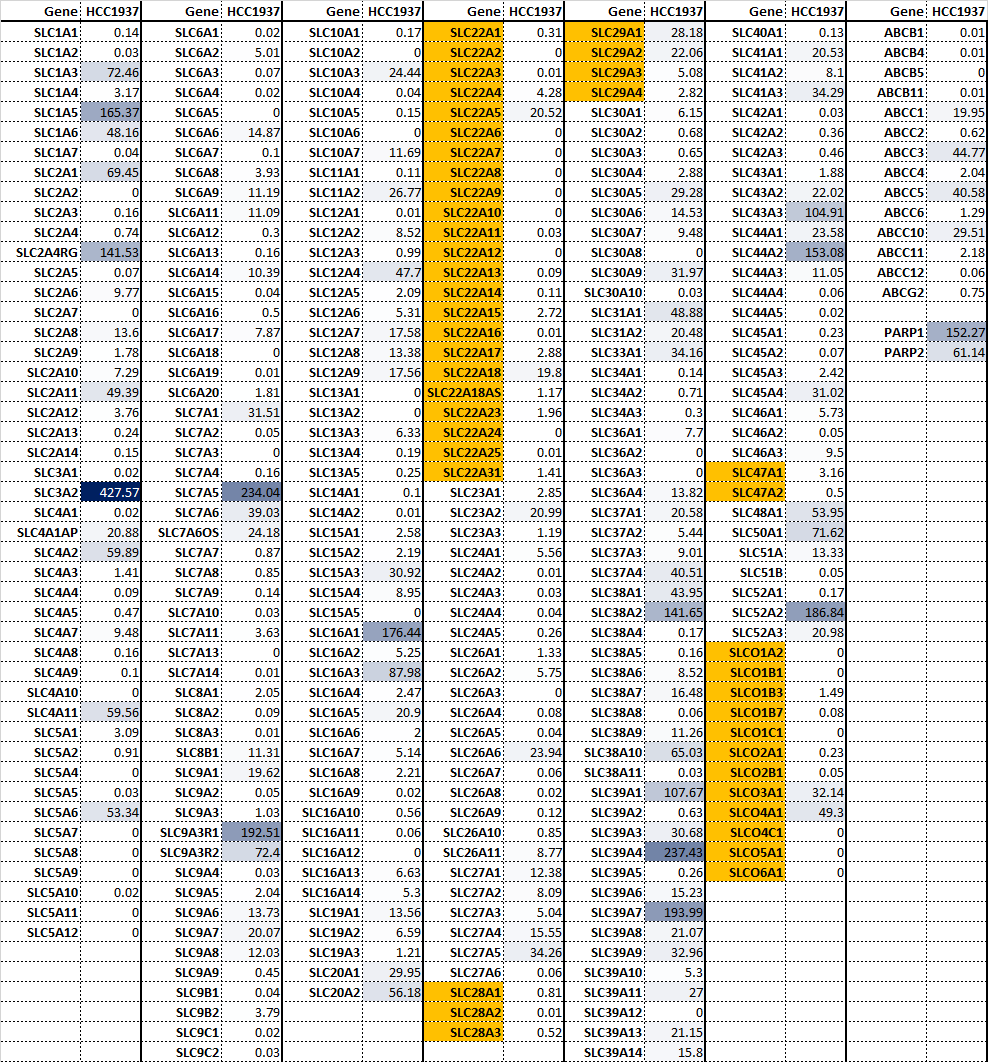


**Supplementary References**

1. Ghandi M, Huang FW, Jané-Valbuena J, Kryukov GV, Lo CC, McDonald ER, et al. Next-generation characterization of the Cancer Cell Line Encyclopedia. Nature. 2019; doi:10.1038/s41586-019-1186-3.

2. Hediger MA, Clemencon B, Burrier RE, Bruford EA. The ABCs of membrane transporters in health and disease (SLC series): Introduction. Mol Aspects Med. 2013;34(2-3):95–107.

3. Nies AT, Koepsell H, Damme K, Schwab M. Organic cation transporters (OCTs, MATEs), in vitro and in vivo evidence for the importance in drug therapy [en]. Handb Exp Pharmacol. 2011; doi:10.1007/978-3-642-14541-4_3.

4. Emami Riedmaier A, Nies AT, Schaeffeler E, Schwab M. Organic anion transporters and their implications in pharmacotherapy. Pharmacol Rev. 2012; doi:10.1124/pr.111.004614.

5. Nies AT, Damme K, Kruck S, Schaeffeler E, Schwab M. Structure and function of multidrug and toxin extrusion proteins (MATEs) and their relevance to drug therapy and personalized medicine. Arch Toxicol. 2016; doi:10.1007/s00204-016-1728-5.

6. Young JD, Yao SY, Baldwin JM, Cass CE, Baldwin SA. The human concentrative and equilibrative nucleoside transporter families, SLC28 and SLC29. Mol Aspects Med. 2013; doi:10.1016/j.mam.2012.05.007.

7. Gessner A, König J, Fromm MF. Clinical aspects of transporter-mediated drug-drug interactions. Clin Pharmacol Ther. 2019; doi:10.1002/cpt.1360.

8. Giacomini KM, Huang SM, Tweedie DJ, Benet LZ, Brouwer KL, Chu X, et al. Membrane transporters in drug development. Nat Rev Drug Discov. 2010; doi:10.1038/nrd3028.

9. Robey RW, Pluchino KM, Hall MD, Fojo AT, Bates SE, Gottesman MM. Revisiting the role of ABC transporters in multidrug-resistant cancer. Nat Rev Cancer. 2018; doi:10.1038/s41568-018-0005-8.

10. Nies AT, Lang T. Multidrug resistance proteins of the ABCC subfamily. In: You G, Morris ME, editors. Drug Transporters: Molecular Characterization and Role in Drug Disposition. John Wiley & Sons, Inc; 2014. pp. 161–85.
